# Short- and long-term modulation of rat prefrontal cortical activity following single doses of psilocybin

**DOI:** 10.1038/s41380-025-03182-y

**Published:** 2025-08-26

**Authors:** Ross J. Purple, Rahul Gupta, Christopher W. Thomas, Caroline T. Golden, Nicola Palomero-Gallagher, Robin Carhart-Harris, Seán Froudist-Walsh, Matthew W. Jones

**Affiliations:** 1 School of Physiology, Pharmacology and Neuroscience, University of Bristol, Biomedical Sciences Building, University Walk, Bristol, UK; 2 School of Engineering Mathematics and Technology, University of Bristol, Merchant Venturers Building, Bristol, UK; 3 Compass Pathways plc, London, UK; 4 Institute of Neuroscience and Medicine (INM-1), Research Centre Jülich, Jülich, Germany; 5 C. and O. Vogt Institute of Brain Research, Medical Faculty, University Hospital Duesseldorf, Heinrich Heine University Duesseldorf, Düsseldorf, Germany; 6 University of California San Francisco, Sandler Neurosciences Center, San Francisco, CA USA

**Keywords:** Neuroscience, Biomarkers

## Abstract

We quantify cellular- and circuit-resolution neural network dynamics following therapeutically relevant doses of the psychedelic psilocybin. Using chronically implanted Neuropixels probes, we recorded local field potentials (LFP) alongside action potentials from hundreds of neurons spanning infralimbic, prelimbic and cingulate subregions of the medial prefrontal cortex of freely-behaving adult rats. Psilocybin (0.3 mg/kg or 1 mg/kg i.p.) unmasked 100 Hz high frequency oscillations that were most pronounced within the infralimbic cortex, persisted for approximately 1 h post-injection and were accompanied by decreased net neuronal firing rates and reduced spike-train complexity. These acute effects were more prominent during resting behaviour than during performance of a sustained attention task. LFP 1-, 2- and 6-days post-psilocybin showed gradually-emerging increases in beta and low-gamma (20–60 Hz) power, specific to the infralimbic cortex. These findings reveal features of psychedelic action not readily detectable in human brain imaging, implicating infralimbic network oscillations as potential biomarkers of psychedelic-induced network plasticity over multi-day timescales.

## Introduction

Convergent evidence suggests that psychedelic-induced ‘reconfiguration’ of brain connectivity patterns can culminate in lasting psychological changes, fuelling substantial interest in psychedelic-based or -derived treatment of mental health problems [1–3]. For example, in a recent phase IIb double blind trial, a single dose of synthesized crystalline polymorph COMP360 psilocybin was shown to significantly reduce depression scores over a period of 3 weeks in people with treatment-resistant depression [4]. However, directly assessing cellular and circuit-level neural mechanisms is challenging in human studies, particularly over multi-day timescales. Defining the molecular, cellular and circuit mechanisms driving psychedelic actions by translating between human brain imaging and animal neurophysiology therefore remains essential [5].

Psychedelics like psilocybin directly modulate the serotonergic (5-HT) system. Psilocybin’s active metabolite, psilocin, is a partial agonist of 5-HT receptors (5-HTRs), primarily acting on 5-HT_2A_, 5-HT_1A_, and 5-HT_2C_ receptors [6, 7]. 5-HT_2A_ and 5-HT_2C_Rs are G_q_-protein coupled receptors (GPCRs) which, on activation, increase cell excitation through the phospholipase C signalling pathway. In contrast, 5-HT_1A_Rs are G_i_-GPCRs which inhibit the activity of adenylate cyclase, leading to decreased cell excitation [5, 8]. Both subtypes are widely expressed throughout rodent, primate and human brains, including in the prefrontal cortex [9–11], which is reciprocally connected to serotonergic nuclei in the brainstem [12]. Immunohistochemical studies in rat have shown both 1 and 2A Rs are expressed in cortical pyramidal cells (1A: 60% of cells, 2A: ~60–90% of cells in PFC) and GABAergic interneurons (1A: 25% of cells, 2A: 25% of cells in PFC) [9]. Thus, while the psychedelic effects of drugs including psilocybin are generally thought to involve activation of 5-HT_2A_Rs [13, 14], other 5-HTR subtypes may contribute to therapeutic potential [15]. Certainly, widespread 5-HTR expression, bidirectional effects on cell excitation, and modulation of both glutamatergic and GABAergic cells makes pinpointing the actions of psilocybin a complex challenge.

Human fMRI and MEG analyses suggest that psychedelics increase neocortical neural signal diversity, an effect associated with desynchronisation within local network populations and desegregation of distributed brain networks [16–20]. Invasive recordings of local field potentials (LFP) can be used to back-translate these analyses into animals, in which a concomitant decrease in low-frequency oscillatory power would be expected following systemic psychedelics. Indeed, psilocybin/psilocin has been shown to decrease low-frequency power in both humans [21, 22] and rodents [23–25]. While studies are few, there is also emerging evidence of the effects of psilocybin on the activity of individual neocortical neurons and their circuit interactions. Golden & Chadderton [23] showed that systemic injection of 2 mg/kg psilocybin led to a net increase in population (mixed pyramidal and interneuron) firing rates in the cingulate cortex of head-fixed mice [23]. More recently, Shao et al. used optogenetic labelling to show that 1 mg/kg psilocybin increased spiking in a minority (20%) of pyramidal tract neurons in the anterior cingulate cortex of mice, while average firing rates in intratelencephalic neurons were less affected [14]. Despite this progress, there is limited knowledge of the effects of psilocybin on large scale cell network activity in freely-behaving animals, though a recent analysis of mouse retrosplenial cortex calcium dynamics showed suppression of fear-associated ensembles following 1 mg/kg psilocybin [26]. Here, we used large-scale, high-density electrophysiology in freely-behaving rats to quantify how psilocybin affects the activities of different cells types at different doses across medial PFC subregions, and how synchrony between cells is affected.

Regarding the long-term effects of psilocybin on underlying brain activity, Daws et al. found decreased brain network modularity post-treatment in two separate samples of individuals treated with psilocybin-therapy for depression, an effect that correlated with improved symptom severity [27]. Two separate analyses on related data found a differential effect of psilocybin-therapy versus the selective serotonin reuptake inhibitor, escitalopram, on brain responses to emotional faces [28] and brain network dynamics [29] supporting the principle that psychedelic-therapy has a different therapeutic action versus conventional antidepressants. Others have shown psilocybin can lead to lasting changes in human resting state functional connectivity [30–32]. On a cellular level, both rodent cell culture and in vivo studies have shown prolonged, psilocybin-induced increases in neuritogenesis and spinogenesis [33, 34]. Identifying the lasting effects of psilocybin on neural network dynamics could bridge between these human and animal findings, linking sustained restructuring of connectivity patterns with potential translational markers of therapeutic effects.

In this study, we therefore quantified the effects of individual systemic injections of therapeutically relevant doses of psilocybin on cellular and network activity recorded from the prefrontal cortex of freely behaving rats. We hypothesised that systemic psilocybin would have heterogenous effects on cell firing rates and alter coordinated activity between neural and interneuronal populations, modulating network state transitions, the complexity of the prefrontal dynamics and attenuating lower-frequency oscillations. In humans, psilocybin’s most pronounced acute and chronic effects on functional connectivity impact the default mode network [32]. We therefore hypothesised that psilocybin’s effects would be more prominent during resting than task-engaged behaviour and include lasting changes in network activity over the 1-week recording period.

## Materials / subjects and methods

### Ethics, animals and surgery

All procedures were performed in accordance with the UK Animals Scientific Procedures Act (1986) and were approved by the University of Bristol Animal Welfare and Ethical Review Board. Six adult male Lister Hooded rats (340–400 g, ~3months old, Charles River UK) were used in this study, though instances of missing data (for example due to varied electrode placement confirmed post-mortem) meant *N* = 4 for some analyses (specified in figure legends). Formal power calculations were not performed and sample size was matched to other studies using Neuropixels to quantify psilocybin’s effects on neocortical firing rates [14]. Rats were housed individually in transparent, high-roofed cages (37 × 31 × 40 cm) enriched with wooden blocks and flowerpots. Water was provided *ad libitum*, with food controlled to maintain body weight at no less than 85% of free-feeding values after recovery from surgery. Rooms were temperature- and humidity-controlled, with lights on at 08:15 and off at 20:15.

2 weeks after delivery, rats underwent stereotaxic surgery under isoflurane recovery anaesthesia, for implantation of Neuropixels 1.0 probes (IMEC, Belgium) into the medial prefrontal cortex (mPFC; AP: +3.25–3.6 mm; ML: 0.7–0.72 mm; DV: −5.5 mm) spanning the infralimbic (IL), prelimbic (PL) and cingulate cortices (CG). Dexamethasone (1 mg/kg i.p.) and a local s.c. injection of the anesthetic Xylocaine 2% with adrenaline into the scalp was given prior to incision. Stainless steel screws (1.1 mm diameter) were placed in the skull contralateral to the probe target location and around the skull for support (glued with Metabond, Sun Medical). A further screw was placed overlying the cerebellum for a ground/reference. For a subset of animals (*n* = 4) an automatic micromanipulator (IVM Mini Single, Scientifica, UK) was used to lower the probe into the brain at a speed of 0.2μm/s with manual lowering at a similar speed in the remaining animals. Dental acrylic with gentamycin (DePuy Synthes, USA) was applied in a first layer over the surface of the skull, with further dental acrylic (Simplex rapid, Kemdent, UK) used for the remainder of the implant construction. A copper mesh cone encircling the implant was cemented around the surface of the skull with a ~3 cm end of a falcon tube cemented on top to contain and protect the electrodes and headstage. A Neuropixels headstage was connected to the probe and cemented to the side of the falcon tube before the lid was screwed on. Buprenorphine (0.05 mg/kg i.p.) was given post-surgery.

### Task protocol

13 ± 4 days after surgery, rats were trained on an operant task for 6–7 days (Supplementary Fig. 1A). On Day 0, rats received an injection of either COMP360 (Compass Pathways’ investigational formulation of synthesized crystalline polymorph psilocybin) or vehicle (saline) counterbalanced across animals, with a 14-day washout period prior to receiving the alternative injection. Psilocybin was dissolved in saline at a concentration of 0.3 mg/ml and the experimenter was not blind to drug condition during recordings. The drug and vehicle were intraperitoneally (i.p.) administered at a volume of 1 ml/kg in counterbalanced order across rats; since all rats underwent all conditions, randomisation for group allocation was not required. The 0.3 mg/kg dose was chosen because it approximates the therapeutic dose used in clinical trials [4] and has been shown to be effective at long-term modulation of affective memory in a rat translational model [35]. However, in a subset of 4 rats a 1 mg/ml solution was additionally administered 1 ml/kg after a further 14-day washout period.

On Day 0, recordings were first taken during a baseline rest and task block before a further 3 rest blocks and 2 task blocks were recorded after injection of the drug (each block was 15 minutes in length). Subsequent recordings were also taken during 2 rest and 2 task blocks on post-injection days 1, 2 and 6 (Supplementary Fig. 1B). All recordings were performed between 08:00–18:00, with drug injections between 09:00–10:00 for rats recorded in the morning, and 14:00–15:00 for rats recorded in the afternoon; recordings started at approximately the same time for each rat across days. Two rats did not have baseline recordings prior to saline injections, so final sample sizes for each condition were Saline: *N* = 4, 0.3 mg/kg: *N* = 6, 1 mg/kg: *N* = 4.

Throughout the injection day and subsequent recording days, single unit activity and local field potentials (LFP) were recorded simultaneously using SpikeGLX software (Karsh et al., Janelia Research Campus). LFPs were sampled at 2.5 kHz; extracellular action potentials were sampled at 30 kHz with a 300 Hz high pass filter. Video monitoring was collected simultaneously throughout the recordings.

At the end of experimental procedures, rats were deeply anaesthetised with sodium pentobarbital and transcardially perfused with 4% paraformaldehyde. 40 µm brain sections were collected and immuno-stained with DAPI, GFAP (Agilent, Z033401-2), and IBA1 (Synaptic Systems, 234308) for staining cell nuclei, astrocytes, and microglia to identify glial scaring around the probe location. Histological images were mapped to a rat brain atlas (Paxinos & Watson, 2007) using the open-source python software HERBS [36]. Identification of the Neuropixels probe tip was then used to interpolate the location of the probe within the prefrontal cortex (Supplementary Fig. 2).

### Analysis of local field potentials and single units

Data were processed in Matlab (Mathworks, MA) and Python (Python Software Foundation). Spectral power density from LFPs was calculated using the MATLAB pwelch function with 4 s windows, no overlap, 0.25 Hz frequency bins. A single LFP channel from each brain region was selected for further analyses (except for Fig. 2). The infralimbic LFP channel was selected as the channel with the highest peak in 100 Hz power within the infralimbic cortex (364 ± 317 µm [mean ± std] from the ventral boundary). Since recorded channels within the prelimbic cortex covered its entire dorsal-ventral boundary for all animals, the prelimbic LFP channel was selected from the approximate centre of the region (808 ± 85 µm from the ventral boundary). The cingulate LFP channel was selected as the highest channel above the ventral boundary which was in an equivalent location across all animals (284 ± 38 µm from the ventral boundary).

For spike data, a common average reference was applied across all channels. Single units were then isolated using automated clustering software (Kilosort 3; Pachitariu et al., 2021) followed by post-processing using the EcEphys pipeline (Allen Institute for Brain Science) and verification using Phy2 (Rossant et al., 2021). For post-processing, only units with inter-spike interval violations <0.5, amplitude cutoff <0.1 and presence ratio >0.9 were selected for further analyses, following default criteria from AllenSDK. Regular-spiking (RS-cells, putative pyramidal cells), narrow-spiking (NS-cells, putative interneurons) and wide-slow spiking cells (WS-cells, putative interneurons) were classified using CellExplorer [37] based on their waveform spike width (<425 μs for narrow interneurons) and tau rise in the autocorrelation (>6 ms for wide interneurons). These classification parameters were fixed and applied to all well-isolated units, independent of recording location or depth.

### Mean-squared displacement and Lempel-Ziv complexity of mPFC dynamics

For each 15 min block, the spike trains of individual units were time-binned using a bin width of ∆*t* = $20$ ms, chosen so the majority of bins contained 0 or 1 spikes, and to avoid over-smoothing of firing rate estimates; qualitatively equivalent results were obtained with 10 ms bins. For binarization, a time-bin was assigned 1 if it had one or more spike counts, and zero otherwise. Thus, the instantaneous state, ${{{\boldsymbol{S}}}}_{{{\boldsymbol{t}}}}$, of a neural network representing the mPFC region at time *t* was defined by a N-bit binary state, ${{{\boldsymbol{S}}}}_{{{\boldsymbol{t}}}}=\left[{s}_{t}^{1},{s}_{t}^{2},\ldots ,{s}_{t}^{N}\right]$, where ${s}_{t}^{i}\in \left\{{\mathrm{0,1}}\right\}$ and $N$ is the number of units recorded within the region. The mean-squared displacement, MSD, of the network state [38] across a time step, $n\Delta t$, ahead is given by,

$${{MSD}}_{n,t}={\left\langle {\left|{{{\boldsymbol{S}}}}_{{{\boldsymbol{t}}}{{\boldsymbol{+}}}{{\boldsymbol{n}}}{{\boldsymbol{\Delta }}}{{\boldsymbol{t}}}}-{{{\boldsymbol{S}}}}_{{{\boldsymbol{t}}}}\right|}^{2}\right\rangle }_{N}$$

For each time *t*, we computed MSD values for multiple time steps of size $n=1-50$. We then averaged the MSD values across n, ${{MSD}}_{t}={\left\langle {{MSD}}_{n,t}\right\rangle }_{n}$ [38].

We pairwise averaged the MSD values across the two post-injection rest and task blocks separately. Pre-drug MSD distributions were z-scored and post-drug distributions were transformed by subtracting the mean of the pre-drug distribution from the post-drug distribution. We then pooled the respective MSD values across rats into a single MSD distribution. Altogether, we had six post-drug pooled MSD distributions for the three drugs (saline, 0.3 mg/kg psilocybin, 1 mg/kg psilocybin) and the two states (rest and task).

To compute the Lempel-Ziv complexity (LZC) of mPFC network dynamics, we binarized the single unit spiking activity data using a time-bin of 10 ms [39, 40]. Thus, for each drug and state, we obtained a unit-wise ordered list of LZC values for the entire mPFC, by pooling the values across all cell types, mPFC sub-regions, and rats. We pairwise averaged the LZC values of the individual units across the two post-drug rest/task states to obtain a single list of post-drug rest/task LZC values for each drug.

### Statistical analyses

All graphs plot means ± S.E.M unless otherwise stated. Boxplots show mean, median, 25 and 75th percentiles. LFP data is shown as an average across animals, single unit data is shown as an average across units. Changes in comparison to baseline were obtained by dividing the average post-injection values by the average baseline values for both drug and saline, conducted separately for rest and task conditions. The change after psilocybin was then divided by the change after saline to calculate percent differences between conditions. Depending on the data type, distributions, and variance, comparisons between saline and psilocybin were assessed using either parametric or non-parametric tests, including all three drug conditions (saline, 0.3 mg/kg, and 1 mg/kg psilocybin) where possible. Differences in behaviour, power density, and coherence were assessed with either a one-way anova (ANOVA1), two-way anova (ANOVA2), a repeated measures anova (RM-ANOVA), or a generalised linear model (GLM). Differences in single unit data were assessed with either a Scheirer-Ray-Hare test (SRH), a Kruskal-Wallis test (KW), a permutation test (PT), or a Chi-squared test (chisq). In all cases, if the test was significant, this was followed by a post-hoc pairwise comparison test (paired and unpaired T-test or Wilcoxon signed rank and rank sum test) followed by a Bonferroni correction (BC) for multiple comparisons. Since analyses of LFP data including power density and coherence analyses involves testing a substantial number of frequency variables (from 0 to 200 Hz at 0.25 Hz resolution), BC was not applied. Instead, a partial Bonferroni correction was used. For this, a principal component analysis was applied to the variable of interest and the number of output principal components that explained over 95% of the variance in the data was used as the alpha correction factor, as previously described (i.e. α = 0.05/number of principle components explaining >95% variance [41]). For the MSD and LZC analyses, Cohens d was calculated to gain insight into the strength of the statistical significance.

## Results

### Psilocybin induces emergence of high-frequency oscillations in infralimbic cortical LFP

**Fig. 1:**
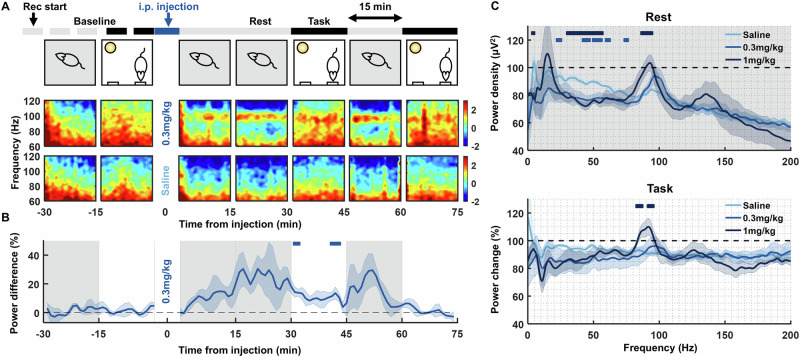
Psilocybin induces frequency-dependent changes in oscillations in medial prefrontal cortical local field potentials (LFP). **A** LFP spectrograms from a single recording channel in the infralimbic cortex of one rat, aligned to rest and operant task 15 min epochs pre- and post-injection of 0.3 mg/kg psilocybin (top row spectrograms) and saline (bottom row). Note the sustained power around 100 Hz during the rest block after injection of 0.3 mg/kg psilocybin. **B** Average time course of ~100 Hz high frequency oscillation 1/f power difference at baseline and post-injection, shown as a difference in power between 0.3 mg/kg psilocybin and saline. Grey shaded and white backgrounds delineate rest and operant task blocks respectively. Bars above trace mark times of significant difference to saline (partial-Bonferroni corrected post-hoc t-tests, *p* < 0.05, *N* = 4). **C** Average change in power density during the rest (top) and task (bottom) blocks from baseline (rest: average from −30 min to −15 min pre-injection, task: from −15 min to 0 min) to post injection (rest: average from 15–30 min and 45–60 min post-injection, task: from 30–45 min and 60–75 min) of saline (*N* = 4), 0.3 mg/kg psilocybin (*N* = 5) and 1 mg/kg psilocybin (*N* = 4). Bars above power change spectra represent significant differences to saline for 0.3 mg/kg psilocybin (lighter blue) and 1 mg/kg (darker blue; partial-Bonferroni corrected post-hoc t-tests, *p* < 0.05).

We chronically implanted Neuropixels probes to record neural activity across the medial prefrontal cortex (mPFC) of freely-behaving rats. Rats performed an operant task in which nose-pokes were rewarded in 15 min blocks (task blocks) interleaved with 15 min, reward-free blocks of resting behaviour (rest blocks; Fig. 1A, Supplementary Fig. 1). Neither 0.3 mg/kg psilocybin, 1 mg/kg psilocybin, nor saline significantly altered movement or behavior based on video tracking or task performance. There was also no evidence of sleep during these recording periods based on local field potentials (LFP). Drug effects on neurophysiology were therefore unlikely to be confounded by modulation of global arousal state, though we cannot rule out subtle changes potentially discernible using other measures.

0.3 mg/kg psilocybin unmasked a sustained ~100 Hz high frequency oscillation (HFO) in infralimbic cortex LFP, evident during both task and rest periods (Fig. 1A). HFO power peaked 15–30 min and lasted at least 60 min post-injection (Fig. 1B; RM-ANOVA: drug: F(1,6) = 5.74, *p* = 0.054, time: F(104,624) = 5.26, *p* < 0.001, drug x time: F(104,624) = 2.63, *p* < 0.001). Power spectra revealed a broadband decrease in power after 0.3 mg/kg psilocybin, except at ~100 Hz. This effect was most prominent during rest blocks (Fig. 1C; Supplementary Fig. 3). Psilocybin also led to significantly decreased 20–80 Hz power compared to saline (ANOVA2 rest blocks: drug: F(2,8010) = 67.04, *p* < 0.001, frequency: F(800,8010) = 7.94, *p* < 0.001; drug x frequency: F(1600,8010) = 0.77, *p* = 1.000). 1 mg/kg psilocybin induced similar changes, albeit with a slowing in the peak of the HFO to ~93 Hz (Fig. 1C).

### Psilocybin-induced HFO centered on the infralimbic cortex

**Fig. 2:**
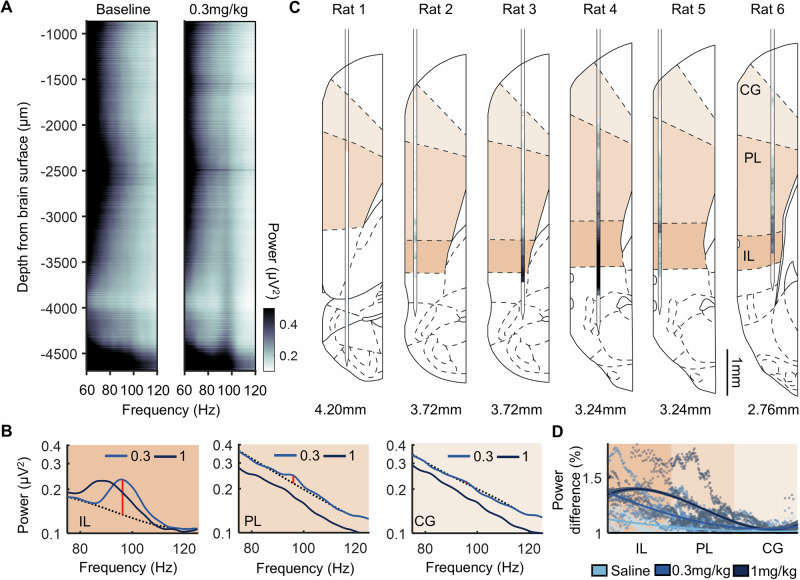
Sustained 80–110 Hz oscillations (HFO) following psilocybin are most pronounced in infralimbic cortex. **A** LFP power between 60–120 Hz across all electrode channels spanning the medial prefrontal cortex of one rat, averaged across the 15 min rest period pre- (left) and post- (right) injection of 0.3 mg/kg psilocybin. **B** Power density from an LFP channel in the cingulate cortex (CG; top), prelimbic cortex (PL, middle) and infralimbic cortex (IL, bottom) during post-injection of 0.3 mg/kg and 1 mg/kg psilocybin. Dotted line denotes interpolated signal. Red line denotes HFO 1/f power difference between raw and interpolated signal. **C** HFO 1/f power difference across all recorded channels for each rat. Darker hues indicate greater difference in HFO power. Value below denotes anterior-posterior stereotaxic coordinate of the probe placement based on post-mortem reconstruction (*N* = 6). **D** HFO 1/f power difference from all LFP electrodes in all rats as a function of their estimated dorsal-ventral location within the prefrontal cortex. Locations were normalised according to the size of each of the three regions assessed. Plot shows data from individual electrodes (dots) and polynomial fits (curves) for each drug condition with shaded areas depicting 95% prediction intervals (*N* = 6).

To determine the anatomical topography of the HFO, spectral power was calculated during post-psilocybin rest blocks across all Neuropixels channels (Fig. 2A). Mapping the 1/f power difference at 80–110 Hz (Fig. 2B) to the histological verification of the Neuropixels placement revealed a continuum of HFO power that was maximal within the infralimbic cortex (Fig. 2C, D). In Rat 1, the probe bypassed the infralimbic cortex; there was no increase in the HFO power discernible in this rat. The strongest increases in HFO power were clearly identified when the probe spanned deeper layers of the cortex (Rats 3, 4, 6 with the probe in layers III, V, VI). In addition to main effects of distance along the probe (β = 0.01, SE = 0.01, t = 2.53, *p* = 0.011, generalised linear model using a log-linked inverse gaussian distribution) and drug (β = 0.20, SE = 0.01, t = 32.07, *p* < 0.001), a significant interaction was also identified between distance and drug condition on HFO 1/f power difference (β = −0.05, SE = 0.01, t = −20.95, *p* < 0.001; Fig. 2D).

### Psilocybin leads to an overall decrease in cell firing rates

**Fig. 3:**
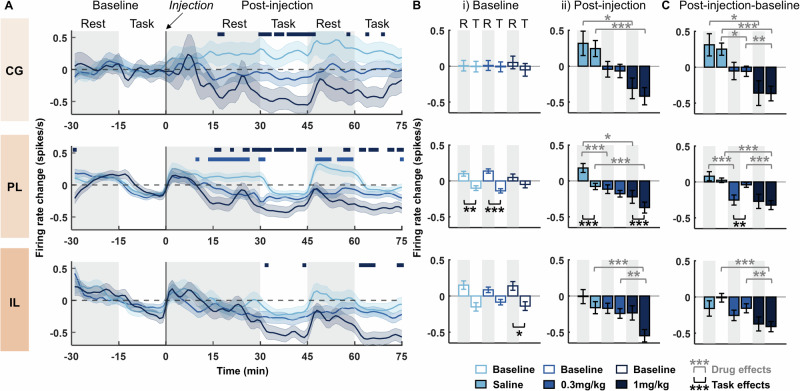
Psilocybin leads to a dose dependent net decrease in RS-cell firing rates that differs across brain regions and behavioural state. **A** Time-course of change in firing rates of RS-cells in the cingulate cortex (CG; top), prelimbic cortex (PL; middle) and infralimbic cortex (IL; bottom) relative to pre-injection baseline (injection at time 0). Firing rates are shown as a difference from the average firing rates during the 30 min pre-injection baseline. Bars above the rate change indicate significant differences of 0.3 mg/kg (light blue) and 1 mg/kg (dark blue) versus saline vehicle injection (post-hoc Bonferroni corrected Wilcoxon rank sum test: *p* < 0.05). Grey shaded and white backgrounds delineate rest and operant task blocks respectively (*N* = 4). **B** Average change in firing rates during rest (R) and task (T) blocks for RS-cells within the CG (top row), PL (middle row) and IL (bottom row). (i) average firing rate change during individual 15 min rest and task blocks at baseline (shown as a difference from average firing rate across the whole 30 min baseline period). (ii) average firing rate change from baseline to post-injection (*N* = 4). **C** Difference in firing rate change between post-injection blocks (shown in Bii) and baseline blocks (shown in Bi), calculated separately for rest (post injection rest – baseline rest) and task (post-injection task – baseline task; *N* = 4). Asterisks denote significant post-hoc Bonferroni corrected Wilcoxon rank sum or signed rank differences between rest and task blocks (signed rank; black) or between drugs (rank sum; grey): ***p < 0.001, **p < 0.01, *p < 0.05. Shaded bands in **A** and error bars in **B, C** denote SEM.

Spike-sorting identified a median of 280 (between 66–669) stable single units per animal during each single ~2 h recording session, with similar numbers of units detected in each drug condition (Supplementary Fig. 4A). 0.3 mg/kg psilocybin significantly decreased RS-cell firing rates within the prelimbic cortex compared to saline. This effect was specific to rest blocks and not apparent when the animal was actively engaged in the task (Fig. 3A), or in cingulate or infralimbic cortex. In contrast, 1 mg/kg psilocybin led to a significant decrease in firing rates in all three brain areas, during both rest and task blocks (Infralimbic: SRH: Drug: H(2,47460) = 245.81, *p* < 0.001, Time: H(104,47460) = 553.96, *p* < 0.001, Drug x Time: H(208,47460) = 425.11, *p* < 0.001; Wilcoxon: *p* < 0.05 (at 1 mg/kg only); Prelimbic: SRH: Drug: H(2,174510) = 779.20, *p* < 0.001, Time: H(104,174510) = 954.46, *p* < 0.001, Drug x Time: H(208,174510) = 583.07, *p* < 0.001; Wilcoxon: *p* < 0.05 (at 0.3 m/kg and 1 mg/kg); Cingulate: SRH: Drug: H(2,22575) = 452.03, *p* < 0.001, Time: H(104,22575) = 99.65, *p* = 0.603, Drug x Time: H(208,22575) = 309.08, *p* < 0.001; Wilcoxon: *p* < 0.05 (at 1 mg/kg only)). Psilocybin did not induce any significant changes in NS-cell firing rates across time or conditions (Supplementary Fig. 4B).

Prelimbic and infralimbic cortical RS-cell firing rates were significantly higher during rest blocks compared to task blocks during baseline conditions (Fig. 3Bi). This behavioral modulation of firing rates was not evident in cingulate cortex, and there were no baseline differences in any brain region across drug conditions (SRH: Cingulate: Task: H(1,430) = 0.33, *p* = 0.568; Drug: H(2430) = 0, *p* = 1.00; Task x Drug: H(2430) = 0.41, *p* = 0.815; Prelimbic: Task: H(1,3324) = 75.80, *p* < 0.001; Drug: H(2,3324) = 0, *p* = 1.00; Task x Drug: H(2,3324) = 13.04, *p* = 0.002; Infralimbic: Task: H(1904) = 21.68, *p* < 0.001; Drug: H(2904) = 0, *p* = 1.00; Task x Drug: H(2904) = 2.94, *p* = 0.230; Wilcoxon: *p* < 0.05). The prelimbic difference between task and rest blocks remained significant post-injection of both saline and 1 mg/kg psilocybin but was diminished after 0.3 mg/kg psilocybin (Fig. 3Bii). All three brain regions showed a significant main effect of drug condition, post-injection (SRH: Cingulate: Task: H(1,430) = 0.30, *p* = 0.583; Drug: H(2430) = 23.74, *p* < 0.001; Task x Drug: H(2430) = 0.79, *p* = 0.674; Prelimbic: Task: H(1,3324) = 9.99, *p* = 0.002; Drug: H(2,3324) = 34.68, *p* < 0.001; Task x Drug: H(2,3324) = 3.98, *p* = 0.137; Infralimbic: Task: H(1904) = 3.71, *p* = 0.054; Drug: H(2904) = 24.10, *p* < 0.001; Task x Drug: H(2904) = 1.57, *p* = 0.456; Wilcoxon: *p* < 0.05).

A comparison of the scale of change in firing rates from baseline to post-injection revealed significantly greater change in firing rates during rest blocks, compared to task blocks, after 0.3 mg/kg psilocybin within the prelimbic cortex (Fig. 3C). This suggests the diminished differences between rest vs. task firing rates after 0.3 mg/kg psilocybin are driven by greater decreases in firing rates during rest blocks. Again, all three brain regions showed a significant main effect of drug condition (SRH: Cingulate: Task: H(1430) = 0. 30, *p* = 0.87; Drug: H(2430) = 32.91, *p* < 0.001; Task x Drug: H(2,430) = 1.97, *p* = 0.373; Prelimbic: Task: H(1,3324) = 2.32, *p* = 0.128; Drug: H(2,3324) = 40.91, *p* < 0.001; Task x Drug: H(2,3324) = 18.54, *p* < 0.001; Infralimbic: Task: H(1904) = 0.02, *p* = 0.890; Drug: H(2904) = 27.56, *p* < 0.001; Task x Drug: H(2904) = 1.04, *p* = 0.594; Wilcoxon: *p* < 0.05).

**Fig. 4:**
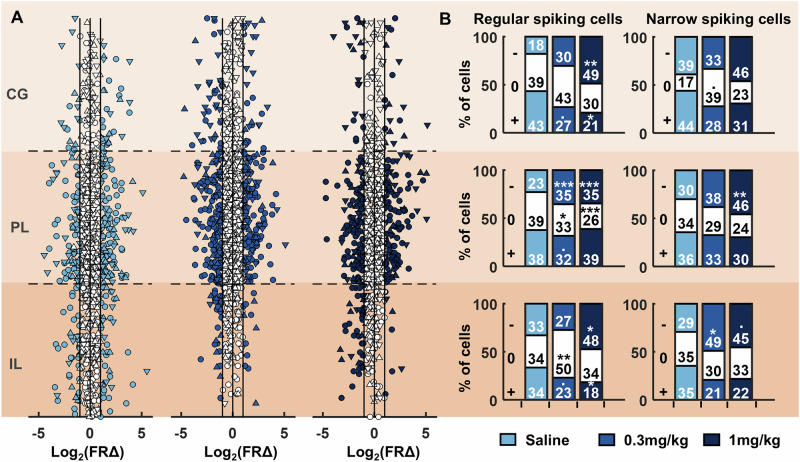
RS-cells and NS-cells of the medial prefrontal cortex respond to systemic psilocybin with heterogeneous changes in firing rate. **A** Individual cell firing rate changes during pre- to post-injection rest blocks sorted according to estimated dorsal-ventral location within the prefrontal cortex. Each of the three brain regions (IL, PL, CG) were normalised in size with approximate location of each cell determined by proximity to electrodes along the probe. Cells coloured in white showed either less than a twofold change in firing rates, or the change did not reach significance. Distributions are shown separately for saline (left), 0.3 mg/kg psilocybin (middle) and 1 mg/kg psilocybin (right). Circles denote RS-cells, triangles pointing up denote NS-cells, triangles pointing down denote WS-cells. **B** Proportion of RS-cells (left) and NS-cells (right) showing a significant decrease (−), increase (+) or no change (0) from baseline to post-injection of psilocybin or saline within each brain region during rest blocks (*N* = 4). Asterisks denote significant post-hoc chi-squared test differences in the proportion of cells between saline and 0.3 mg/kg or 1 mg/kg psilocybin; ***p < 0.001, **p < 0.01, *p < 0.05, •*p* < 0.05 but did not survive Bonferroni multiple comparison correction.

Population-level averages masked heterogenous changes in the firing rates of individual RS- and NS-cells across injection conditions (Fig. 4A). To detail this heterogeneity, we next compared the proportion of RS- and NS-cells across regions that showed significant changes in firing rates pre- to post-injection, revealing a significantly higher proportion of RS-cells within the infralimbic and prelimbic cortex that had either decreased or no change in firing rates post-injection of 0.3 mg/kg psilocybin, compared to saline (Fig. 4B; chisq: saline vs 0.3 mg vs 1 mg; infralimbic decrease: X^2^ = 11.98, *p* = 0.003; infralimbic no change: X^2^ = 10.13, *p* = 0.006; infralimbic increase: X^2^ = 10.03, *p* = 0.007; prelimbic decrease: X^2^ = 26.05, *p* < 0.001; prelimbic no change: X^2^ = 18.61, *p* < 0.001; prelimbic increase: X^2^ = 8.60, *p* = 0.014; post-hoc chisq, *p* < 0.05). There were no differences in the proportion of cells changing firing rates in the cingulate cortex between 0.3 mg/kg psilocybin and saline (chisq: saline vs 0.3 mg vs 1 mg; cingulate decrease: X^2^ = 11.88, *p* = 0.003; cingulate no change: X^2^ = 2.17, *p* = 0.338; cingulate increase: X^2^ = 7.94, *p* = 0.019; post-hoc chisq, *p* > 0.05). Following 1 mg/kg psilocybin, the proportion of cells with decreased post-injection firing rates was significantly higher in all three brain regions, in comparison to saline (post-hoc chisq, *p* < 0.05).

In general, a greater proportion of NS-cells decreased firing rate in both infralimbic and prelimbic cortices, but this only reached significance in the infralimbic cortex at 0.3 mg/kg psilocybin and in the prelimbic cortex at 1 mg/kg psilocybin in comparison to saline, after controlling for multiple testing (chisq saline vs 0.3 mg vs 1 mg; infralimbic decrease: X^2^ = 7.15, *p* = 0.028; infralimbic no change: X^2^ = 0.43, *p* = 0.808; infralimbic increase: X^2^ = 5.08, *p* = 0.079; prelimbic decrease: X^2^ = 8.72, *p* = 0.013; prelimbic no change: X^2^ = 4.47, *p* = 0.107; prelimbic increase: X^2^ = 1.03, *p* = 0.598; cingulate decrease: X^2^ = 1.57, *p* = 0.456; cingulate no change: X^2^ = 6.12, *p* = 0.047; cingulate increase: X^2^ = 2.91, *p* = 0.233; post-hoc chisq, *p* < 0.05).

### Psilocybin reduces the complexity of mPFC network activity

**Fig. 5:**
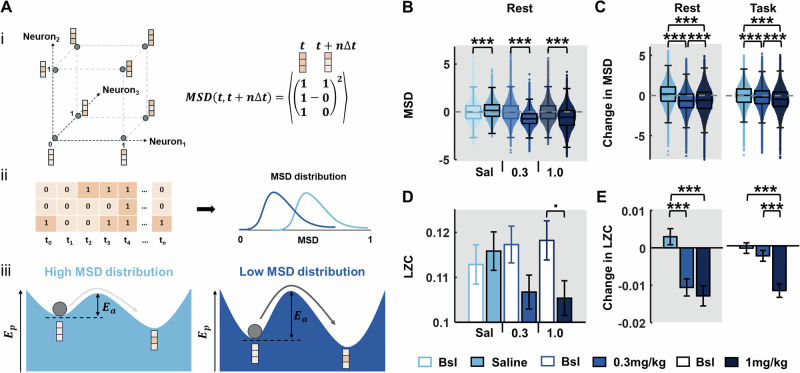
Psilocybin leads to decreased mean square displacement (MSD) of neural spiking activity and decreased Lempel-Ziv complexity (LZC). **A** The spiking activities of recorded single units in the mPFC can be used to describe the binary state transitions of a representative network of the units: (i, left) An example three-neuron network is shown, with possible states visualised as the vertices of a unit cube. The possible 3-bit binary states, where 0 (light-orange) represents no spikes in the time bin, and 1 (dark-orange) represents spiking. The axes represent the binary values of the individual neurons. The neural network (dark-grey ball) transitions from one state to another with time. (i, right) The MSD is a Euclidean jump distance between any two network states over a discrete time-step ∆t. (ii) The temporal dynamics of the network are shown as consecutive 3-bit states at discrete time points, t_1_ to t_n_. At every time point, an average MSD of the network transitions over the next 1 s is computed, giving a distribution of MSDs characterising network transitions across the entire time duration. MSD distributions centred on lower values reflect restricted state transitions, while higher values indicate relatively free network state transitions. (iii) Schematic descriptions of the potential energy landscapes, E_p_, for the low and high MSD distributions are shown. Shallower landscapes (left) require less activation energy leading to more frequent state transitions (light grey arrow) and a high MSD distribution. Network states sitting in a deep basin of attraction (right) requires larger activation energy, E_a_, to jump to neighbouring states, causing less-frequent transitions (dark grey arrow) and low MSD distribution. **B** MSD from all single units across pre- and post-injection rest blocks for each drug condition, indicating a decrease in MSD under psilocybin. **C** Change in MSD from pre- to post-injection rest blocks (left) and task blocks (right), across drug conditions, indicating a dose-dependent decrease in MSD with psilocybin. **D** LZC from all single units across pre- and post-injection rest blocks for each drug condition. **E** Change in LZC from pre- to post-injection rest blocks (left) and task blocks (right), across drug conditions, indicating a decrease in LZ complexity with psilocybin (0.3 and 1 mg/kg) in both rest and task contexts. Error bars denote SEM (*N* = 4). Asterisks denote significant post-hoc t-test Bonferroni corrected differences between saline and 0.3 mg/kg or 1 mg/kg psilocybin; ***p < 0.001, •*p* < 0.05 but did not survive Bonferroni multiple comparison correction.

To quantify mPFC network dynamics, time-binned spikes from all units were binarized and the mean-squared displacement (MSD) was calculated to describe the scale of network state transition across time (Fig. 5A). This analysis revealed a significant increase in MSD from pre- to post- injection of saline, in contrast to a significant, dose-dependent decrease after psilocybin (Fig. 5B, C; KW: Rest blocks: saline: X^2^(1,359598) = 3357.1 *p* < 0.001, 0.3 mg/kg: X^2^(1,359598) = 46604.58 *p* < 0.001, 1 mg/kg: X^2^(1,359598) = 26416.76 *p* < 0.001). Consistent with LFP and firing rate analyses, psilocybin’s effects were larger during rest periods (Cohen’s d, Rest blocks: saline vs 0.3 mg/kg: d = 1.04, saline vs 1 mg/kg: d = 0.81) than task periods (Task blocks: saline vs 0.3 mg/kg: d = 0.27, saline vs 1 mg/kg: d = 0.54).

Less frequent network state transitions lead to lower entropy activity [42]. We therefore computed Lempel-Ziv complexity (LZC) as a measure of entropy in neural spiking [39]. While there were no significant differences pre- to post-saline, a significant decrease in LZC was observed after both 0.3 mg/kg and 1 mg/kg psilocybin (Fig. 5D; PT, one-tailed: saline: *p* = 0.3131, 0.3 mg/kg: *p* = 0.0280, 1 mg/kg: *p* = 0.0148; PT, two-tailed: saline: *p* = 0.6260, 0.3 mg/kg: *p* = 0.0595, 1 mg/kg: *p* = 0.0304). Pair-wise subtracting the units’ LZC values during the pre-drug rest/task blocks from values during the post-drug rest/task blocks showed that 0.3 mg/kg psilocybin led to a significant decrease in complexity compared to saline during rest, but not task blocks (Independent 2-sample t-test: rest *p* < 0.001, task *p* = 0.3096). 1 mg/kg psilocybin led to a significant decrease in complexity compared to saline during both rest and task blocks (Fig. 5E; Independent 2-sample t-test: rest *p* < 0.001, task *p* < 0.001). Collectively, these results suggest that psilocybin leads to more ordered and less chaotic mPFC activity, by deepening the underlying energy landscape of mPFC dynamics, hence restricting wide and frequent network state transitions.

### Long-term effects of psilocybin on coordinated oscillations in the infralimbic cortex

**Fig. 6:**
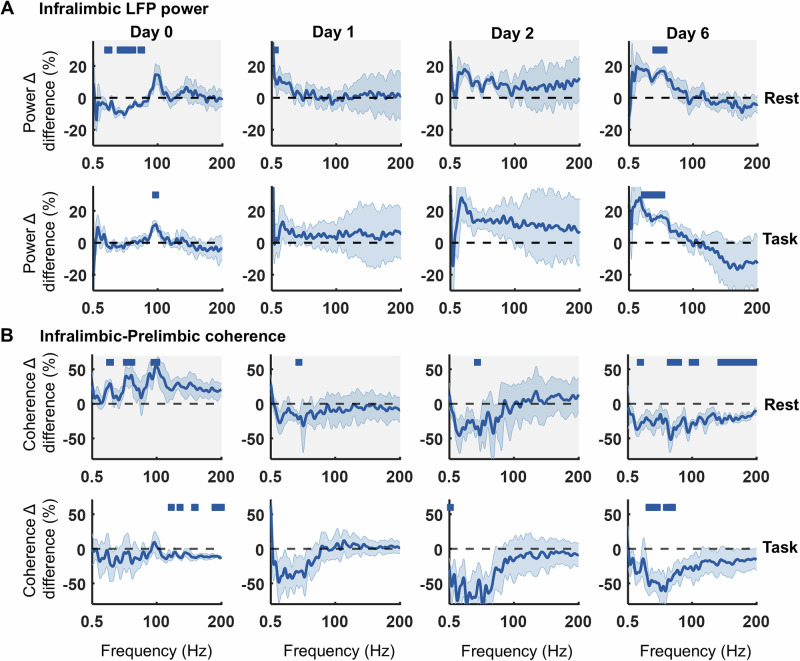
A single injection of psilocybin induces long-term changes in LFP power in infralimbic cortex across 6 days post-injection. **A** Difference between 0.3 mg/kg psilocybin in comparison to saline in the change in power density from baseline to post injection during rest (top, grey) and task (bottom, white) blocks, on the day of injection (Day 0) and subsequent days 1, 2 and 6 (all compared to day 0 baseline, *N* = 4). **B** Difference between 0.3 mg/kg psilocybin in comparison to saline in the change in infralimbic-prelimbic coherence from baseline to post injection across days during rest (top, grey) and task (bottom, white) blocks (all compared to day 0 baseline, *N* = 4). Bars indicate significant differences between 0.3 mg/kg psilocybin and saline (partial-Bonferroni corrected post-hoc t-tests, *p* < 0.05). Shaded bands denote SEM.

To identify potential long-term effects of low-dose psilocybin, recordings on Day 0 were compared with drug-free activity on post-injection days 1, 2 and 6. The post-injection infralimbic HFO was an acute effect of psilocybin and absent from day 1 onwards. However, across days there was a gradual increase in power between 20–60 Hz during both rest and task blocks (Fig. 6A, Supplementary Fig. 5; ANOVA2: Rest Day 6: Frequency: F(800,4806) = 7.4, *p* < 0.001; Drug: F(1,4806) = 177, *p* < 0.001; Frequency x Drug: F(800,4806) = 1.24, *p* < 0.001; Task Day 6: Frequency: F(800,4806) = 0.48, *p* = 1.000; Drug: F(1,4806) = 28.94, *p* < 0.001; Frequency x Drug: F(800,4806) = 1.31, *p* < 0.001). This psilocybin-induced evolution of 20–60 Hz power across days was not evident for LFPs in the prelimbic or cingulate cortices (Supplementary Fig. 6).

On Day 0, a broad increase in coherence between pairs of LFP channels was identified across all frequencies following 0.3 mg/kg psilocybin during rest blocks but a significant decrease in coherence >100 Hz during task blocks (Fig. 6B, shown for IL-PL, with similar findings for IL-CG and PL-CG; ANOVA2: IL-PL, Rest Day 0: Frequency: F(800,4806) = 7.86, *p* < 0.001; Drug: F(1,4806) = 2176.07, *p* < 0.001; Frequency x Drug: F(800,4806) = 0.73, *p* = 1.000; IL-PL, Task Day 0: Frequency: F(800,4806) = 1.05, *p* = 0.186; Drug: F(1,4806) = 427.64, *p* < 0.001; Frequency x Drug: F(800,4806) = 0.28, *p* = 1.000). Similar results were found for both power and coherence at 1 mg/kg dose of psilocybin in comparison to saline (Supplementary Fig. 7). These coherence patterns evolved across Days 1–6, gradually decreasing at frequencies below 100 Hz and culminating in a broadband decrease in coherence by day 6 (Fig. 6B, shown for IL-PL LFPs; ANOVA2: Rest Day 6: Frequency: F(800,4806) = 2.38, *p* < 0.001; Drug: F(1,4806) = 1744.14, *p* < 0.001; Frequency x Drug: F(800,4806) = 0.34, *p* = 1.000; Task Day 6: Frequency: F(800,4806) = 0.61, *p* = 1.000; Drug: F(1,4806) = 1809.94, *p* < 0.001; Frequency x Drug: F(800,4806) = 0.59, *p* = 1.000; ANOVA2 results for day 1 and 2 can be found in Supplementary Table 1).

## Discussion

We show that single, systemic injections of psilocybin modulate network activity across the rat medial prefrontal cortex, with most effects centred on the infralimbic subregion during resting behaviour. Psilocybin induced a mix of firing rate changes across different putative cell types but led to dose-dependent net decreases in the firing rates of both RS- and NS-cells (likely reflecting pyramidal cell and interneuron populations respectively), particularly during rest periods when animals were disengaged from the task. This net suppression of spiking activity was accompanied by a slowing of spiking dynamics and a reduction in their complexity. Local field potentials revealed the infralimbic cortex as the epicentre of an acute ~100 Hz high-frequency oscillation, and the emergence over several days of persistently increased 20–60 Hz power and decreased intra-cortical coherence.

### Acute effects of psilocybin on oscillatory network activity

Psilocybin has previously been shown to acutely decrease low-frequency network oscillations, potentially reflecting desynchronisation of cortical networks, decreased top-down inhibitory control and consequent modulation of distributed cognitive processing [1, 5]. While 2 mg/kg psilocybin (i.p.) decreased delta power within the cingulate cortex in mice [23] and 10 mg/kg psilocybin (i.v.) reduced theta power in rat EEG [43], we show only marginal decreases in low frequency power immediately following the 1 mg/kg dose. We also identified acute decreases in LFP low gamma power after both 0.3 mg/kg and 1 mg/kg psilocybin. Our results are consistent with psilocybin-induced broadband decreases in power over frontal areas previously shown in rats [25] and humans [16, 44].

Analyses of psilocybin’s effects on distributed functional connectivity based on extracranial recordings of human MEG [44] and rat EEG [25, 43] have tended to identify decreased functional connectivity at low frequencies. In contrast, we identified acute, broadband increases in LFP coherence between subregions of the mPFC after 0.3 mg/kg psilocybin. Binding occupancy of psilocybin to 5-HT2ARs is around 21% at 0.2 mg/kg and 41% at 1 mg/kg in rats [45], hence the increased broadband intracortical coherence following the lower 0.3 mg/kg dose in our study could reflect modulation of more frontally-localized neural circuits at lower receptor occupancies.

Consistent with this suggestion, psilocybin’s acute induction of sustained, 100 Hz HFO appeared to be centered on the infralimbic cortex. A recent study described similar HFO in rat EEG following i.v. infusion of 1 or 10 mg/kg psilocybin, though the topography of those HFO was not reported [43]. While these HFO are reminiscent of those induced by dissociative NMDA receptor channel blockers including ketamine, MK801 and phencyclidine [46, 47], the biophysical basis of psilocybin-induced HFO remains enigmatic. HFO were not accompanied by net increases in population firing rates, and it remains to be established whether the ~20% of RS- and NS-cells that increased spiking activity following psilocybin may contribute to increased HFO power.

5-HT_2A_-R agonists and antagonists can induce increases and decreases in HFO power respectively, prompting a suggestion that HFOs may be modulated by 5-HT_2A_Rs [48]. However, pyramidal and interneuronal expression levels of 5-HT_2A_Rs are lower in infralimbic cortex than either prelimbic or cingulate cortices [49], while all three subregions express similar levels of 5-HT_1A_Rs (with highest expression in layer VI). Preferential induction of HFO by psilocybin in the infralimbic cortex may therefore reflect proportionally greater activation of 5-HT_1A_Rs at low doses. Indeed, the infralimbic region corresponds to the cortical region of highest 5-HT_1A_R expression in rats, macaque monkeys and humans [10]. As this is not true for 5-HT_2A_Rs [10, 50], psilocybin’s effects on infralimbic mPFC could be more strongly driven by 5-HT_1A_R effects than in other parts of cortex. This contrasts with other subregions of rodent mPFC, for instance mouse anterior cingulate cortex, where recent evidence strongly implicates 5-HT_2A_Rs in activation of pyramidal tract projection neurons by psilocybin [14]. These gradients of serotonin receptor densities across the cortex, with relatively higher 5-HT_1A_R expression in infralimbic mPFC, hint at nuanced mechanisms through which top-down control may be modulated by psilocybin.

### Acute effects of psilocybin on cellular activity

Until now, very few studies quantified the effects of psilocybin on neural spiking in-vivo. Golden & Chadderton previously identified acutely increased population firing rates within the mouse anterior cingulate cortex after 2 mg/kg psilocybin [23], while other studies have assessed the effects of DOI, another 5-HT_2_ agonist, with mixed findings of either increased [51, 52] or decreased [53–55] neuronal activity within the rodent mPFC. Wood et al. suggest these inconsistencies could be explained by the brain area and layer recorded from – studies that showed an increase after DOI were primarily from recordings of prelimbic and infralimbic layer V. In contrast, and similarly to our results, studies showing a decrease in firing after DOI were more heterogeneous in brain area and layers recorded [55]. In addition, since DOI is not a significant agonist of 5-HT_1A_Rs, our results also likely reflect the action of psilocybin on both 5-HT_1A_ and 5-HT_2A_ receptors.

There are several potential mechanisms for psilocybin-induced decreases in the spiking activity of pyramidal neurons. The subcellular distribution of 5-HT receptors suggests that the predominance of inhibitory 5-HT_1A_Rs on somatic, peri-somatic and axon initiation segments (AIS) might supress neural spiking, while a high density of 5-HT_2A_Rs on distal dendrites can lead to stronger synaptic excitations and dendritic signal reception [56]. Increased 5-HT_2A_R-dependent activation of potassium M-currents in mouse mPFC has also been reported to decrease the neuronal spiking through increased spike frequency adaptation and a depolarization block [57].

Although the net decreases in firing rate identified in our study belie a heterogenous mix of firing rate changes in individual cells, they suggest that psilocybin may lead to net suppression of the PFC’s influence over cognition during the drug’s acute influence.

### Psilocybin-induced slowing and constraining of mPFC dynamics and influence

fMRI/MEG/EEG studies have reported increased LZ complexity of brain dynamics as an empirical characteristic of altered consciousness in humans under psychedelic drugs, including psilocybin, LSD and DMT [18, 58–62]. Psychedelic-enhanced LZ complexity is also associated with more frequent recurrent state transitions in fMRI signals [42]. These studies mainly dealt with cortex-wide dynamics and low spatiotemporal resolution metrics of neural activity [18]. Translating between fMRI and MEG/EEG signals primarily originating from slower time-scale, synaptic dynamics and our analysis of psilocybin’s effects on mPFC spiking dynamics is complex. Nevertheless, our finding of decreased spiking complexity acutely following psilocybin was unexpected, and inconsistent with the human literature. The ‘relaxed beliefs under psychedelics’ (REBUS) hypothesis states that top-down signal transmission along the cortical hierarchy is reduced while the bottom-up information transfer significantly increases [63]. Decreased mPFC neural spiking, network state transitions and LZ complexity may contribute to such flattening of the global cortical hierarchy by reducing top-down drive.

### Delayed emergence of elevated gamma rhythms

Preclinical studies have found that psychedelics promote anatomical neuroplasticity such as dendritic spinogenesis in cortical pyramidal neurons which has been linked to enduring behavioural changes [64]. The most prominent finding from our longitudinal data was a gradual increase in beta and slow gamma-frequency activity, that was highest on day 6 post-injection of 0.3 mg/kg psilocybin. Gamma oscillations are generated by rhythmic activity in interconnected inhibitory-inhibitory or excitatory-inhibitory loops [65], particularly involving fast-spiking parvalbumin (PV)-positive interneurons and their rhythmic entrainment of pyramidal neurons with feedback inhibition [5, 66, 67]. The gradual increase in gamma shown in our study may therefore reflect psilocybin-induced plasticity in these microcircuits, ultimately leading to an alteration to E/I balance. Within the limited existing data on long-term effects of psilocybin, a change in gamma activity has not been investigated. In a study of resting state functional connectivity in 10 psychedelic naïve volunteers, McCulloch et al. identified decreased connectivity within the prefrontal cortex 1 week (but not 3 months) after a ~ 0.3 mg/kg dose of psilocybin [30]. Siegel et al. also identified persistent decreases in hippocampal functional connectivity up to 21 days post-psilocybin [32]. Similarly, we found long-term decreases in broadband coherence across the CG/PL/IL cortices. This, combined with the finding of increased gamma power, corroborate previous findings of psychedelics inducing cortical desynchronisation [5, 16].

### Limitations and conclusions

One challenge with analysing these data arose from changes in mPFC neurophysiology following saline vehicle injections, most likely reflecting effects of stress from the injection procedure. Since the injection procedure was consistent across drug and vehicle conditions and the order was randomised across animals, our analyses control for these effects. Nevertheless, lower-stress injection methods without restraint of the animal would be useful for future studies [68, 69], as would high-resolution analyses of behavior and arousal (for example using pupillometry). In addition, all experiments reported here were run during the rats’ inactive (lights on) phase. Serotonergic signalling modulates, and is modulated by, circadian processes; future rodent and human work may therefore benefit from comparisons of psychedelic effects on brain dynamics across different circadian and sleep/wake phases. Finally, combining high-density neurophysiology with pharmacological and/or genetic manipulations to pinpoint which of psilocybin’s targets mediate the effects of systemic injection would clearly be of great relevance to translational and clinical investigations of psychedelics.

We have identified that a systemic injection of psilocybin leads to localised, state-dependent changes to the medial prefrontal cortex of rats. Our findings of decreased spectral power at low frequencies, increased power above 80 Hz, decreased cell firing rates, and local slowing of spiking dynamics, add subregional and cellular resolution to previous findings of psilocybin inducing desynchronisation across the brain [32]. Several of these effects were most prominent during disengaged rest periods, consistent with prior human MEG findings [70] and clinical trial practice in which psilocybin is administered in low-stimuli environments. In addition, the localised infralimbic HFO could represent a novel biomarker of psychedelic action. Optogenetic modulation of infralimbic activity can recapitulate the enduring effects of ketamine on structural plasticity and behaviour [71] and, in humans, Brodmann area 25 (the closest homolog to infralimbic cortex in rats [72]) has been associated with hyperactivity in treatment-resistant depression. A decrease in activity in this area has also been associated with clinical response to both antidepressant medication and deep brain stimulation [73]. Our results complement these findings, provoking further investigation into the infralimbic cortex and its circuitry as therapeutic targets preferentially modulated by psilocybin or more selective derivatives.

## Supplementary information

Supplemental Material

## Data Availability

All spike times and analysis code are openly available at https://osf.io/t69ap/ and https://github.com/RahulGupta23019/PsiloDynamics.git.
