# Correction: Short- and long-term modulation of rat prefrontal cortical activity following single doses of psilocybin

**DOI:** 10.1038/s41380-025-03231-6

**Published:** 2025-09-05

**Authors:** Ross J. Purple, Rahul Gupta, Christopher W. Thomas, Caroline T. Golden, Nicola Palomero-Gallagher, Robin Carhart-Harris, Seán Froudist-Walsh, Matthew W. Jones

**Affiliations:** 1 School of Physiology, Pharmacology and Neuroscience, University of Bristol, Biomedical Sciences Building, University Walk, Bristol, UK; 2 School of Engineering Mathematics and Technology, University of Bristol, Merchant Venturers Building, Bristol, UK; 3 Compass Pathways plc, London, UK; 4 Institute of Neuroscience and Medicine (INM-1), Research Centre Jülich, Jülich, Germany; 5 C. and O. Vogt Institute of Brain Research, Medical Faculty, University Hospital Duesseldorf, Heinrich Heine University Duesseldorf, Düsseldorf, Germany; 6 University of California San Francisco, Sandler Neurosciences Center, San Francisco, CA USA

**Keywords:** Neuroscience, Biomarkers

Correction to: *Molecular Psychiatry* 10.1038/s41380-025-03182-y, published online 26 August 2025

In this article the author’s name Robin Carhart-Harris was incorrectly written as Robin Carhartt-Harris. The original article has been corrected.

