# Supplementary material for: Short- and long-term modulation of rat prefrontal cortical activity following single doses of psilocybin
Source: Mol Psychiatry. 2025 Aug 26;30(12):5889–900. doi: 10.1038/s41380-025-03182-y (PMC12602310; doi:10.1038/s41380-025-03182-y)
Supplement: Supplementary file 1 — Supplemental Material [file 41380_2025_3182_MOESM1_ESM.pdf]

Short- and long-term modulation of rat prefrontal cortical activity following single doses of psilocybin

Ross J. Purple PhD, Rahul Gupta PhD, Christopher W. Thomas PhD, Caroline T. Golden PhD, Seán Froudist-Walsh PhD, Nicola Palomero-Gallagher PhD, Robin Carhartt-Harris PhD, Matthew W. Jones PhD

Supplementary Materials

This document provides supplementary figures including results of operant behaviour, histological verification of probe location, time course of narrow-spiking cell firing rates, and longitudinal drug effects on prelimbic and cingulate cortex LFP.

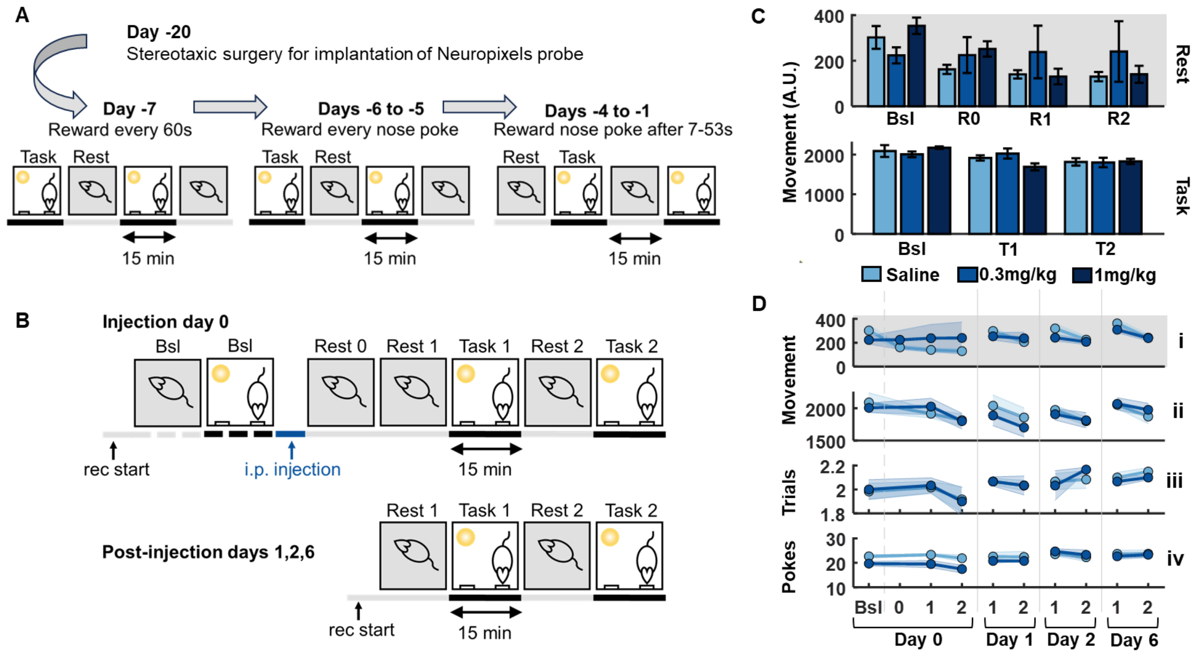

Supplementary Figure 1: *Activity and operant behaviour are not disrupted by systemic injection of 0.3mg/kg or 1mg/kg psilocybin. (A) Training on the operant task began approximately 2 weeks after chronic implantation of the Neuropixels probe into the medial prefrontal cortex (N=6 rats). Initial training sessions lasted for 1h: two 15min task blocks with the house light on and nose poke holes open, interleaved with two 15min rest blocks with the house light off and nose poke holes closed. On the first training day (day -7), ~0.2ml of 30% sucrose solution liquid reward was dispensed automatically every 60s during the task block. On days -6/-5 a fixed ratio of one nose poke for one reward was applied. On days -4 to -1 animals performed the full operant task with the reward released upon nose poke after a random interval between 7-53s. The order of rest/task blocks was also switched. Rats were tethered from day -5 onwards to acclimatise to the electrophysiological recoding setup.* ***(B)*** *On the first injection day (Day 0) rats completed a 30min baseline session comprised of a 15min rest and 15min operant task block. Rats were then injected i.p. with either 0.3mg/kg psilocybin or saline vehicle (counterbalanced across animals), and again completed another operant task session with one initial 15min rest block (to account for initial drug effects) followed by a further four 15min blocks of alternating rest and task epochs (total 1hour 15min). Recordings were repeated on days 1, 2 and 6 with a 1h block of the operant task. 14 days after the first injection, rats received the alternative psilocybin/saline injection, and the protocol was repeated. A further 14 days after this, rats received a 1mg/kg injection of psilocybin, and the protocol was repeated.* ***(C)*** *Behavioural activity during day of injection. Bars show*

average video-recorded movement (a.u.) for each 15-minute block during rest (top) or task (bottom) blocks (Bsl=pre-injection baseline block). ***(D)*** *Behavioural performance on the operant task across sessions during and following 0.3mg/kg psilocybin or saline. (i) Total activity (a.u.) during each rest block on the day of injection and days 1, 2 and 6 post-injection. (ii) Total activity (a.u.) during each task block across days. (iii) Average number of trials completed per minute for each task block. (iv) Average number of nose pokes to the rewarded hole per minute for each task block. Video-recorded movement pre- to post-injection revealed similar activity levels after injection of either saline, 0.3mg/kg psilocybin, or 1mg/kg psilocybin during either task or rest blocks (ANOVA1: Rest blocks: F(5,18)=2.1, p=0.113; Task blocks: F(5,18)=2.53, p=0.067). On post-injection days 1, 2 and 6, performance of the operant task remained similar following 0.3mg/kg psilocybin and saline injections, whether quantified using number of trials completed, nose pokes, or gross activity (all RM-ANOVA p values >0.05). Consistent performance on the task following vehicle and psilocybin injections therefore enabled analyses of drug effects during both task-focused and resting states. Note that recordings were taken on the day of injection of 1mg/kg psilocybin injection, but not on days 1-6 after injection. N=4.*

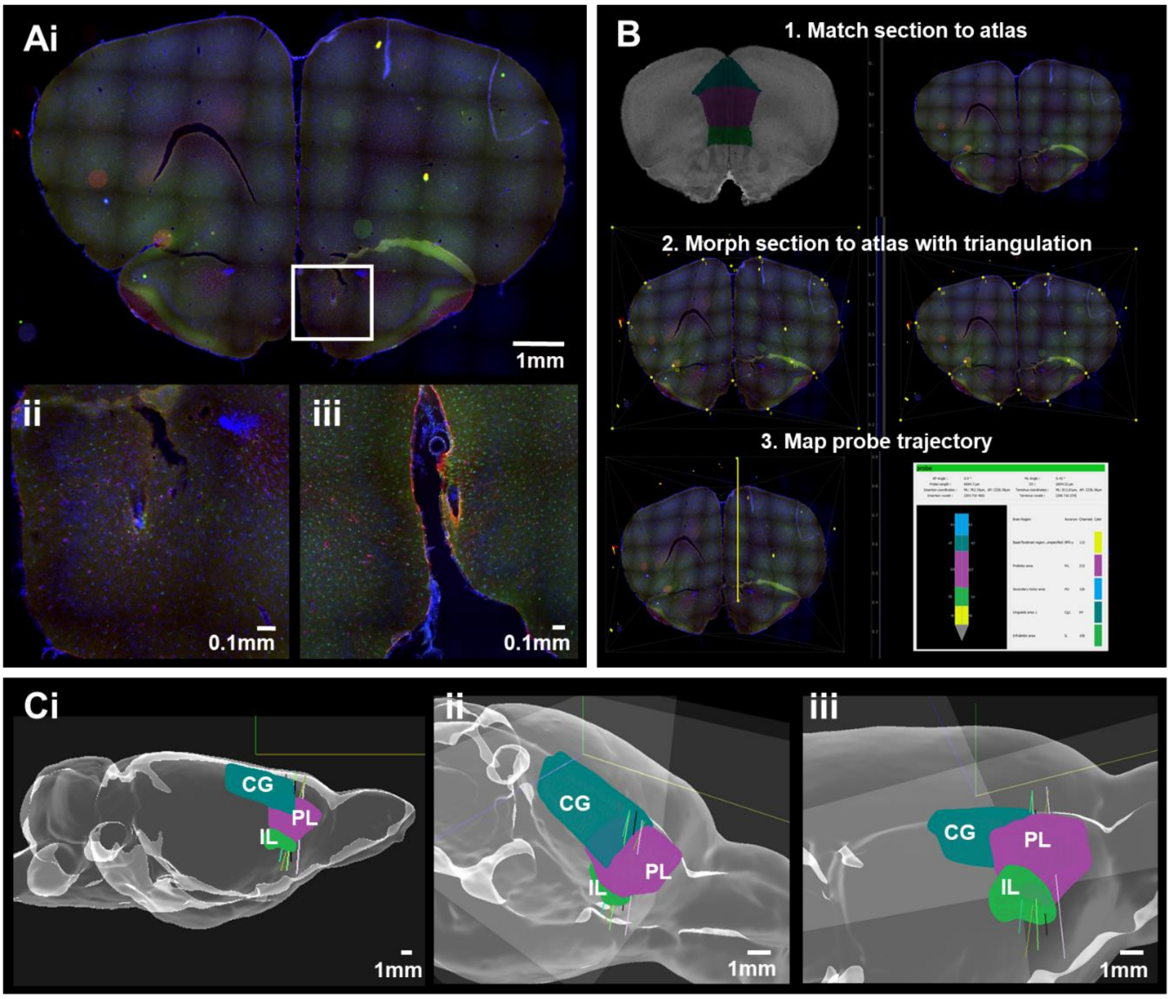

Supplementary Figure 2: *Anatomical identification of the Neuropixels probe.* ***(A)*** *(i) 50um coronal brain slices were stained for glial markers (GFAP, IBA1) to identify scarring from Neuropixels probe. Sections containing scarring from the probe tip (centre of white square) were identified. (ii) Magnification of white square from section in panel A, showing scarring from the probe tip. (iii) A second example of probe tip from a separate rat.* ***(B)*** *Sections were processed using HERBS (Fuglstad et al., 2023) to map the probe trajectory within the brain.* ***(C)*** *(i) Sagittal,*

(ii) Dorsal-ventral, and (iii) ventral-dorsal views of all Neuropixels probes implanted within the medial prefrontal cortex (CG=cingulate cortex, PL=prelimbic cortex, IL=infralimbic cortex).

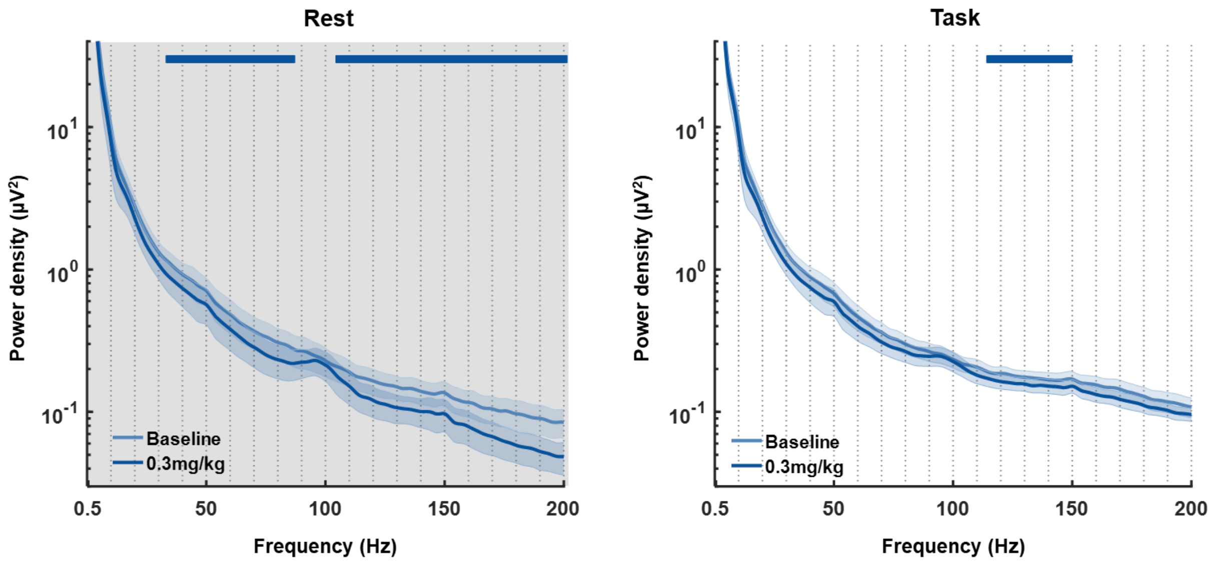

Supplementary Figure 3. *Average power density from an infralimbic cortex LFP channel during a baseline and post-0.3mg/kg psilocybin injection rest block (left) and operant task block (right). Bars above trace represent significant differences between pre- and post-injection of 0.3mg/kg psilocybin (partial-Bonferroni corrected post-hoc t-tests, p<0.05). N=5.*

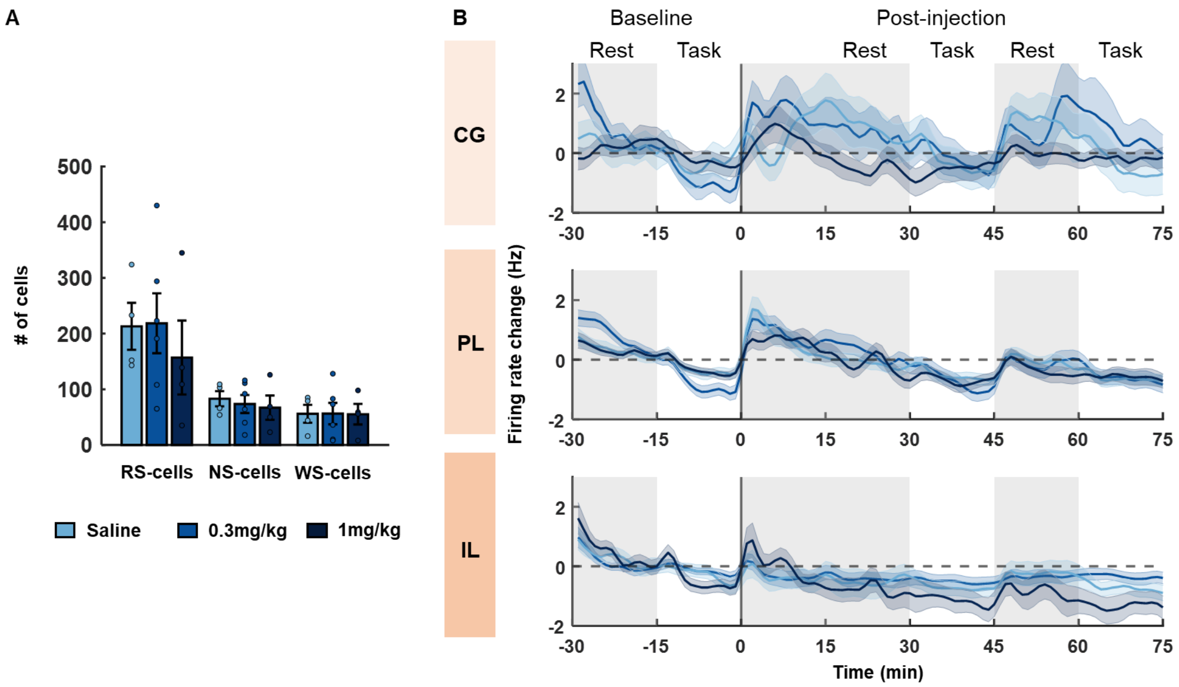

Supplementary Figure 4: (A) *Total number of single cells identified during recordings on the day of injection of psilocybin or saline, separated into regular spiking (RS-cells), narrow spiking (NS-cells) and wide-slow spiking (WS-cells)* ***(B)*** *Time-course of change in firing rates of NS-cells in the cingulate cortex (CG; top), prelimbic cortex (PL; middle) and infralimbic cortex (IL; bottom) from pre-injection baseline. Firing rates are shown as a difference to the average firing rates during the 30-minute pre-injection baseline. Grey shading reflects rest blocks, white reflects task blocks. No differences were identified between conditions.*

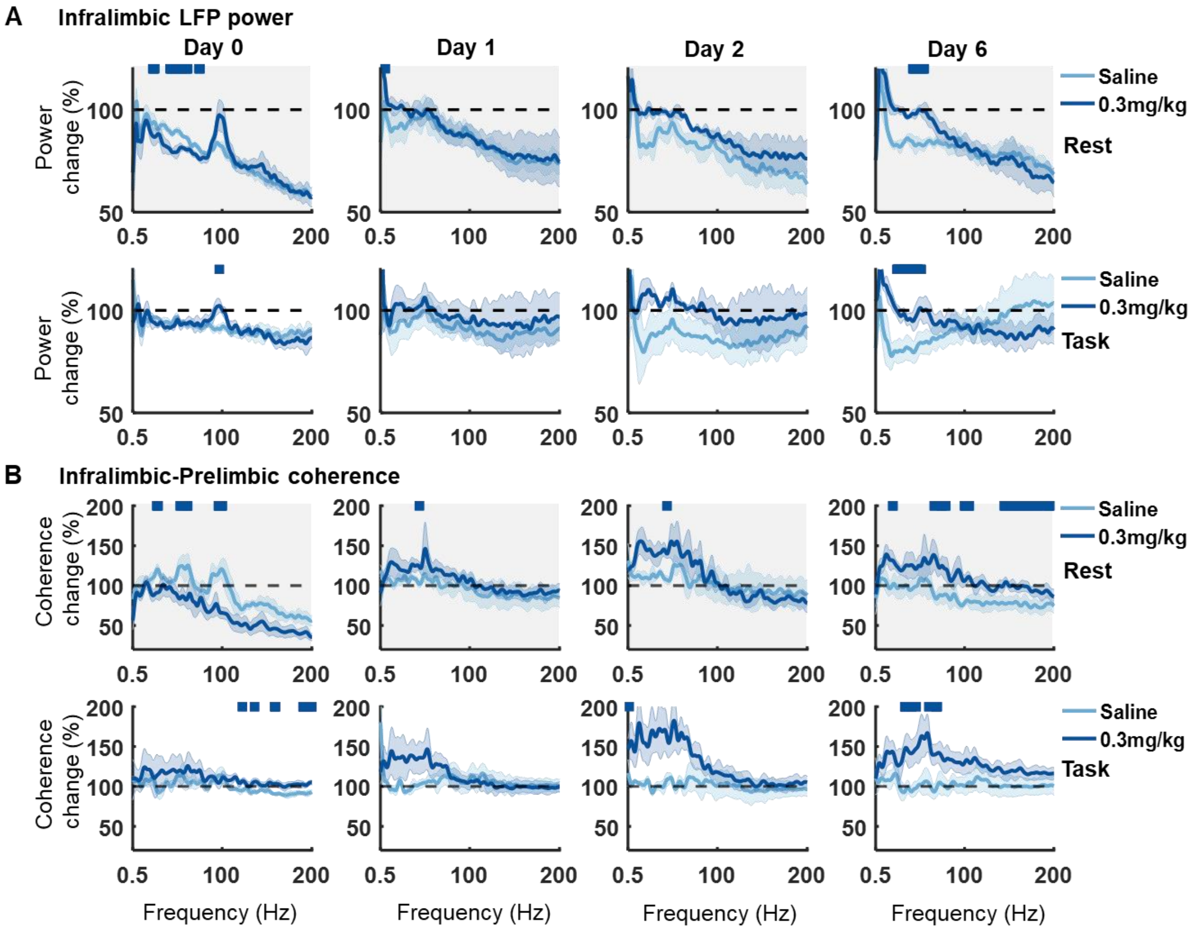

Supplementary Figure 5: ***(A)*** *Change in power density from baseline to post injection of saline (light blue) and 0.3mg/kg psilocybin (dark blue) during rest (top, grey) and task (bottom, white) blocks, on the day of injection (Day 0) and subsequent days 1, 2 and 6 (all compared to day 0 baseline).* ***(B)*** *Change in infralimbic-prelimbic coherence from baseline to post injection of saline and 0.3mg/kg psilocybin across days during rest (top, grey) and task (bottom, white) blocks (all compared to day 0 baseline). Bars indicate significant differences between 0.3mg/kg psilocybin and saline (partial-Bonferroni corrected post-hoc t-tests, p<0.05). Shaded bands denote SEM.*

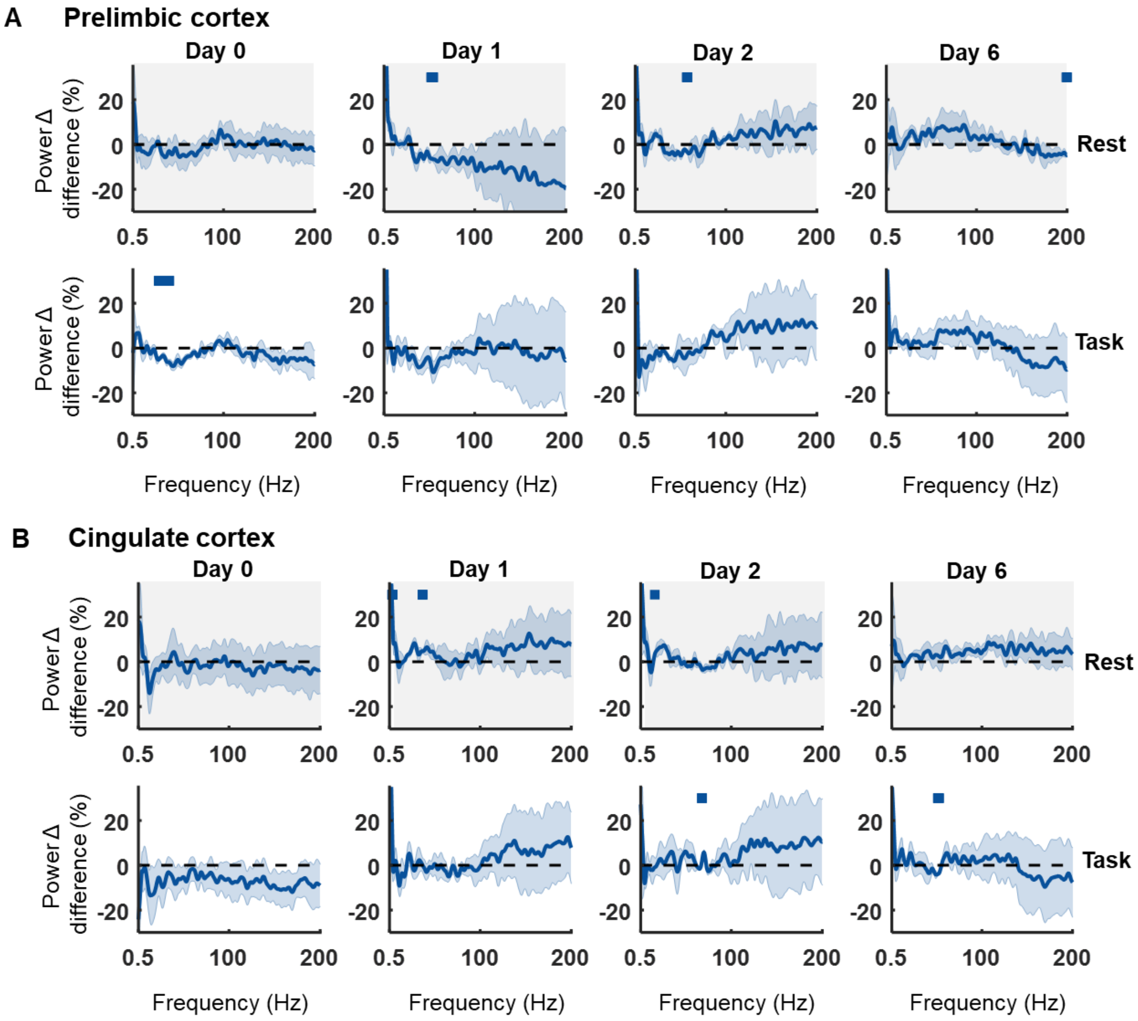

Supplementary Figure 6: (A) *Difference between 0.3mg/kg psilocybin in comparison to saline in the change in power density within the prelimbic cortex from baseline to post injection during rest (top, grey) and task (bottom, white) blocks, on the day of injection (Day 0) and subsequent days 1, 2 and 6 (all compared to day 0 baseline).* ***(B)*** *Same but for LFPs in the cingulate cortex. Bars indicate significant differences between 0.3mg/kg psilocybin and saline (partial-Bonferroni corrected post-hoc t-tests, p<0.05). Shaded bands denote SEM.*

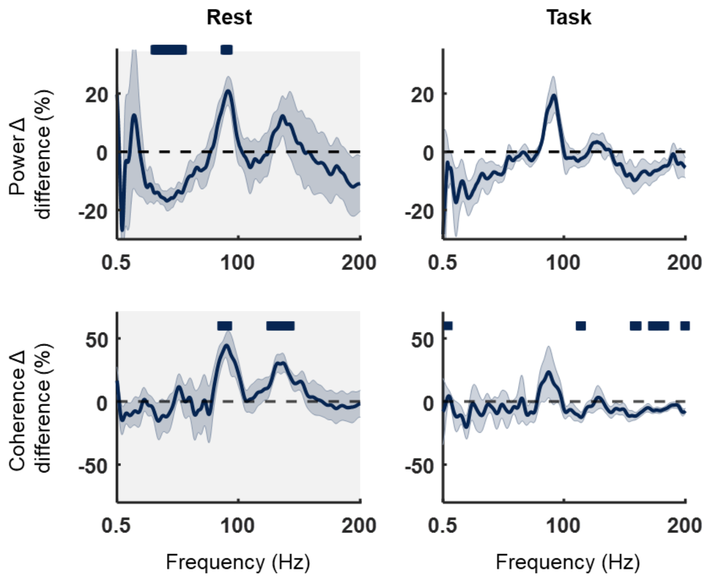

Supplementary Figure 7: *Acute effects of 1mg/kg psilocybin on LFP power and coherence. (Top) Difference between 1mg/kg psilocybin in comparison to saline in the change in power density from baseline to post injection during rest (top, left) and task (top, right) blocks, on the day of injection (Day 0). (Bottom) Difference between 1mg/kg psilocybin in comparison to saline in the change in infralimbic-prelimbic coherence from baseline to post injection rest (bottom, left) and task (bottom, right) blocks. Bars indicate significant differences between 1mg/kg psilocybin and saline (partial Bonferroni corrected post-hoc t-tests, p<0.05).*

Supplementary Table 1: *Two-way analysis of variance results for difference between saline and 0.3mg/kg psilocybin in the change in power and coherence from pre-injection baseline to post-injection day 1 and 2. N=4.*

Block	Day	Factor 1		Factor 2		Interaction	
Power		Frequency		Drug		Frequency x Drug	
Rest	1	F(800,4806)=3.37	p<0.001	F(1,4806)=67.98	p<0.001	F(800,4806)=0.36	p=1.000
Task	1	F(800,4806)=0.64	p=1.000	F(1,4806)=143.90	p<0.001	F(800,4806)=0.45	p=1.000
Rest	2	F(800,3204)=3.60	p<0.001	F(1,3204)=613.30	p<0.001	F(800,3204)=0.16	p=1.000
Task	2	F(800,3204)=0.52	p=1.000	F(1,3204)=566.35	p<0.001	F(800,3204)=0.14	p=1.000
Coherence		Frequency		Drug		Frequency x Drug	
Rest	1	F(800,4806)=2.03	p<0.001	F(1,4806)=264.56	p<0.001	F(800,4806)=0.27	p=1.000
Task	1	F(800,4806)=0.95	p=0.847	F(1,4806)=159.96	p<0.001	F(800,4806)=1.06	p=0.120
Rest	2	F(800,3204)=2.69	p<0.001	F(1,3204)=113.85	p<0.001	F(800,3204)=0.76	p=1.000
Task	2	F(800,3204)=1.93	p<0.001	F(1,3204)=1352.7	p<0.001	F(800,3204)=1.41	p<0.001
